# Dupilumab-induced generalized pustular psoriasis in an elderly patient: a case report and literature review^؆^

**DOI:** 10.1016/j.abd.2026.501378

**Published:** 2026-06-13

**Authors:** Sangho Lee, Francis Yi Xing Lai, Senhong Lee

**Affiliations:** a Department of Dermatology, Monash Health, Clayton, VIC, Australia; b Skin Health Institute, Carlton, VIC, Australia; c Department of Dermatology, Eastern Health, Box Hill, VIC, Australia; d School of Clinical Sciences, Faculty of Medicine, Nursing and Health Sciences, Monash University, Clayton, VIC, Australia

Dear Editor,

Phenotypic switching from atopic dermatitis to psoriasis is a significant adverse effect of dupilumab.^1^ Cases of dupilumab-induced Generalized Pustular Psoriasis (GPP) have been reported but remain rare.^2^, ^3^, ^4^, ^5^, ^6^, ^7^

An 82-year-old male presented with an acute onset of generalized pustular eruption two weeks after starting treatment with dupilumab. He had a two-year history of generalized adult-onset eczema with a baseline EASI of 26.4. His eczema had been confirmed on histopathology, which showed spongiosis with focal eosinophilia, and had only partially responded to topical corticosteroids. He did not report joint pains, fevers, rigors, or preceding coryzal symptoms. He did not have a history of psoriasis or previous smoking, and there were no other recent changes to his medications.

Examination revealed well-demarcated erythematous plaques with overlying pustules affecting the abdomen, back and all four limbs (Fig. 1A). Psoriasiform plaques were also seen on the scalp, extending beyond the frontal hairline. There were coalescing pustules over palmar surfaces (Fig. 1B) but no oral mucosal involvement or palpable lymphadenopathy. The patient remained with stable vital signs and no fever.

**Figure 1:**
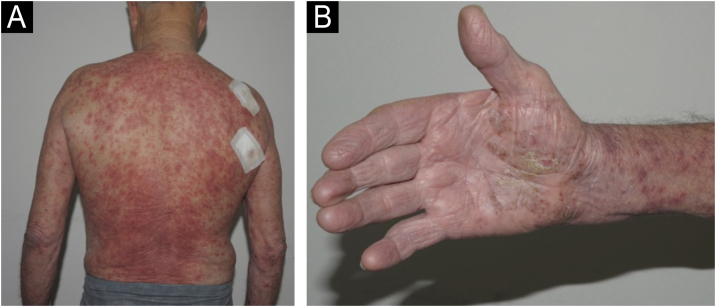
(A) Well-demarcated erythematous plaques with overlying pustules affecting the back and upper limbs (abdomen and lower limbs not shown). (B) Coalescing pustules over the bilateral palmar surfaces.

Histopathology demonstrated psoriasiform hyperplasia with Kogoj’s spongiform pustules, consistent with pustular psoriasis (Fig. 2). Swabs of pustules were negative for bacterial, fungal and viral studies. Full blood examination was unremarkable aside from mild neutrophilia and eosinophilia. His blood tests showed no evidence of immunosuppression, autoimmune diseases, or malignancies, with calcium levels and serum protein electrophoresis also within the normal range.

**Figure 2:**
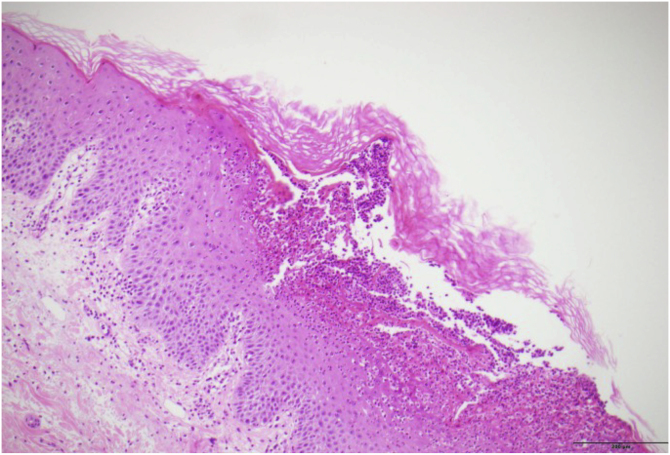
Histopathology findings demonstrating features in keeping with pustular psoriasis, with evidence of psoriasiform hyperplasia and Kogoj spongiform pustule (Hematoxylin & eosin ; ×10).

A diagnosis of dupilumab-induced GPP was made. Dupilumab was discontinued and the patient was treated with intensive topical therapy and oral cyclosporine 175 mg twice daily (5 mg/kg/day). Cyclosporine was gradually reduced over six months, and methotrexate 15 mg weekly was initiated. His rash improved significantly within two weeks after starting treatment, and the response remained at follow-up three years later.

Dupilumab is a human monoclonal IgG4 antibody targeting the Interleukin (IL)-4 Receptor subunit α (IL-4Rα).^1^ This inhibits IL-4/13 signaling pathways that mediate T-helper-2 cell (Th-2) immune response involved in Atopic Dermatitis (AD).^1^ Psoriasis is driven by Th1/Th17 cell response with increased Tumor Necrosis Factor-α (TNF-α), IL-17, and IL-23 levels. A widespread psoriasiform eruption caused by dupilumab, such as GPP or erythrodermic psoriasis, remains rare.^8^

A literature review on Medline, Embase and PubMed identified six cases of histologically confirmed dupilumab-induced pustular psoriasis (Table 1).^2^, ^3^, ^4^, ^5^, ^6^, ^7^ The median age of patients, including our case, is 22-years, and the symptoms began one day to three months after starting dupilumab. All but one patient developed erythematous plaques with pustulosis affecting multiple body areas. All cases resolved promptly within a median time of two weeks following topical or systemic treatments and cessation of dupilumab. Three cases received systemic immunosuppression with corticosteroids or oral cyclosporine, and two received oral antibiotics, but without confirmation of bacterial infections.

**Table 1:** Summary of literature review of dupilumab-induced pustular psoriasis.

Author & year	Age at onset, gender	Ethnicity	Onset of lesions after dupilumab	Distribution	Clinical features	Histology	Bacterial culture performed?	Treatment	Time to resolution
Jia et al, 2022 ^2^	23, M	Chinese	10 days	Lower legs	Superficial pustules on erythematous papules and plaques, worsening after second dose	Parakeratosis, hyperkeratosis, epidermal hyperplasia, dilated capillaries, and lymphocyte infiltrate in the upper dermis; dense neutrophil infiltration within stratum corneum and subcorneal zone forming spongiform abscesses	Yes - negative	Cyclosporine (unclear if intravenous (IV) or oral) and topical steroids	2 weeks
Uysal et al, 2022 ^3^	22, M	Not specified	3 months	Dorsal hands	Hyperkeratotic erythematous plaques with sharp and irregular margins, confluent yellow pustules within plaques	Regular, psoriasiform acanthosis and parakeratosis with elongated rete ridges and reduced stratum granulosum; perivascular lymphocytic infiltrate in the upper dermis; subcorneal spongiotic neutrophilic pustules	No	Topical potent corticosteroid and topical calcipotriol	6 weeks
Zhong et al, 2022 ^4^	51, F	Chinese	3 weeks	*Generalized*	Prior to dupilumab treatment: disseminated erythema papules and scaling with multiple small sterile pustules and crust, worsening 1-week after second injection	Parakeratosis, psoriatic hyperplasia, and dilated tortuous vessels in the papillary dermis; neutrophilic and perivascular lymphocytic infiltration in the superficial dermis	No	IV methylprednisolone 40 mg/day for 1-week, transitioned to weaning course of oral prednisolone	3 weeks
Liu et al, 2023 ^5^	13, M	Not specified	*Post* first dose	Trunk and lower extremities	Diffuse erythema, papules, scales, and mossy plaques with localized vesicles, scattered pustules and exudation	Epidermal hyperkeratosis with fused dyskeratosis, neutrophilic micro-abscesses in the dyskeratotic stratum corneum, reduced to absent granular layer beneath the dyskeratosis, hypertrophy of the spinous layer, upwardly displaced dermal papillae, dilated capillaries, superficial perivascular lymphocytes, and scattered neutrophil infiltration	No	“Anti-allergic” and topical moisturising emollient (unclear which anti-allergic agent used)	Not specified
Dang et al, 2024 ^6^	4, M	Not specified	1 week	Neck, shoulder, back, chest, bilat armpits, groin, buttocks	Erythema and papules with pruritus, followed by extensive, painful desquamation; white pinhead-sized pustules and red papules. Periodic recurrence of pustules and desquamation	Acanthosis, mild spongiosis, subcorneal neutrophil-rich pustules, hyperkeratosis, decreased granular layer, slight perivascular lymphocytic infiltrate in the upper dermis and neutrophils in dermis	No	Topical desonide and mometasone furoate creams and oral desloratadine, followed by oral prednisolone 1 mg/kg/day for two weeks all with limited effect; commenced on oral thiamphenicol 0.25 g twice a day (BD) for two weeks then slowly tapered	2 weeks post commencement of oral thiamphenicol
Liu et al, 2024 ^7^	3.5, M	Not specified	1 day post initial dupilumab dose, 1 day post re-challenge three months later	*Generalized* – including left elbow and abdomen	On initial introduction of dupilumab: generalized erythematous plaques and pustules, fever with congestion in both eyes. On rechallenge: similar cutaneous symptoms without fever or conjunctival congestion	Hyperkeratosis and parakeratosis, abscess formation in the stratum corneum, local crust formation, acanthosis thickening with mild spongy oedema, and chronic inflammatory cell infiltration around small vessels in the superficial dermis	No	Oral sulfoxamycin 0.5 g/day, topical halometasone, topical tacrolimus, topical hydrocortisone butyrate ointment, topical mupirocin	Initially 1 week; rechallenged 2 months post with recurrence, resolved after 1 month of cessation
Lee et al, 2026	82, M	Caucasian	2 weeks	Abdomen, back, bilateral upper and lower limbs including palms	Well-demarcated erythematous plaques with overlying pustules, psoriasiform plaques on the scalp extending beyond the frontal hairline; coalescing pustules over palmar surfaces	Psoriasiform hyperplasia, thickened epidermis with intra-epithelial polymorphs, basal spongiosis and very prominent subcorneal/upper intra-epidermal pustule formation	Yes – negative	Topical corticosteroids, oral cyclosporin 175 mg BD (5 mg/kg/day)	2-weeks

The immune mechanisms of AD and psoriasis exist on polar ends of the T-cell spectrum. IL-4 acts directly on T-cells, dendritic cells and keratinocytes to inhibit IL-23 production and the downstream Th17 polarisation.^8^ Therefore, IL-4 inhibition by dupilumab can induce a shift from Th2-polarised atopic response to a psoriasis-like, Th17/Th1-dominant pattern.^1^ Increased IL-36 expression is also important in dupilumab-induced GPP.^8^, ^9^ The secretion of IL-36 by keratinocytes induces neutrophil infiltration and pustule formation.^9^ IL-36 interacts reciprocally with Th17 cytokines by regulating Th17 differentiation and maintenance, but also is upregulated by the Th17 cytokines such as TNF-α and IL-17.^9^ This immunological interplay can be exacerbated by IL-4/IL-13 signaling blockade, predisposing patients to GPP.^8^, ^9^

A differential diagnosis for generalized pustular eruption following drug use is Acute Generalized Exanthematous Pustulosis (AGEP).^10^ GPP patients typically present with concurrent psoriasiform plaques and frequently extracutaneous symptoms such as arthritis.^10^ AGEP occurs acutely after administration of a causative drug and has a predilection for flexural areas.^10^ AGEP resolves with cessation of offending agent and supportive management, whereas GPP often requires systemic treatment such as oral acitretin or immunosuppressants.^10^ Of note, three cases in our review showed rapid resolution with cessation of dupilumab and topical therapy only, a clinical course more typical for AGEP.

In conclusion, dupilumab can trigger phenotypical transformation to GPP, and clinicians must remain vigilant to the development of generalized erythematous plaques with pustules post-dupilumab use.

## ORCID ID

Francis Yi Xing Lai: 0000-0003-3976-1432

Senhong Lee: 0000-0003-0246-0484

## Research data availability

Does not apply.

## Financial support

None declared.

## Authors' contributions

Sangho Lee: Data collection, or analysis and interpretation of data; writing of the manuscript or critical review of important intellectual content; data collection, analysis and interpretation; critical review of the literature.

Francis Yi Xing Lai: The study concept and design; effective participation in the research guidance; final approval of the final version of the manuscript.

Senhong Lee: The study concept and design; effective participation in the research guidance; final approval of the final version of the manuscript.

A formal written consent has been obtained from the patient for the publication of the case and the use of the clinical photographs.

## Conflicts of interest

Dr Sangho Lee has no conflict of interest to declare. Dr Francis Yi Xing Lai has received grants from Johnson and Johnson, Pierre Fabre and Leo Pharma. Dr Francis Yi Xing Lai is an honorary investigator at Abbvie, Amgen, BMS, Evelo Bioscience, Johnson & Johnson, Leo Pharma and Sunpharma. Dr Francis Yi Xing Lai serves as an honorary member of the advisory board for Bristol Myers Squibb, Johnson and Johnson, L’Oreal, Pierre Fabre and Leo Pharma. Dr Francis Yi Xing Lai served as an honorary speaker for Abbvie, Amgen, Bristol Myers Squibb, Eli Lilly, Galderma, Johnson and Johnson, L’Oreal, Pierre Fabre, Leo Pharma, Novartis, Sanofi and UCB. Dr Senhong Lee has received grants from Johnson and Johnson. Dr Senhong Lee is an investigator for Johnson and Johnson, Abbvie and Amgen. Dr Senhong Lee serves as an honorary member of the advisory board for Abbvie and Bristol Myers Squibb. Dr Senhong Lee has served as an honorary speaker for Johnson and Johnson, UCB, Arrotex and Bristol Myers Squibb.
